# On the Functions of the Roots of the Spinal Nerves. Physiological and Pathological Researches for Estimating the Doctrines of Bell

**Published:** 1845-04

**Authors:** 


ART. V.—*Ueber die Verrichtung der Wurzeln des Rückenmarksnerven, &c.* Von J. W. ARNOLD.—*Heidelberg*, 1844.

*On the Functions of the Roots of the Spinal Nerves. Physiological and Pathological Researches for estimating the Doctrines of Bell.* By J. W. ARNOLD.—*Heidelberg*, 1844. 8vo, pp. 142.

WE formerly introduced Dr. Arnold to our readers as the critic of the reflex theory of Dr. Hall; in the present volume, he appears as the critic of the doctrines of C. Bell. Dr. Arnold introduces his subject by a historical notice of the reception of these doctrines by physiologists, and of their study and progress. Then in eleven chapters he details successively the views of Bell *in extenso*, and the results of inquiries and vivisections by Magendie, Bellingeri, Schoeps, Backer, Langenbech, Müller, Seubert, Panizza, Van Deen, and an anonymous writer in Roser and Wenderlich's *Archiv.* In the two remaining chapters he describes researches of his own as to the functions of the anterior and posterior roots of the spinal nerves, and institutes a comparison between the latter and the cerebral nerves.

We gather from these reviews and from the author's own conclusions that he is of opinion the anterior roots give off muscular and not motor nerves—the posterior, cutaneous and not sensitive nerves exclusively. The anterior nerves endow the muscles with sensation as well as motion. The posterior roots are the channels of the external impressions made on the surface; they constitute the organ of the sense of touch, are, in fact, like the cerebral sensual nerves. On the contrary, the muscular nerves are not suited to receive external impressions; their sensibility is of a specific character, and has reference exclusively to the condition of the muscles. They are not the media through which chemical, mechanical, or galvanic irritants act on the sensorium, but they communicate to the latter the changes which occur in the muscular fibre. They are, of course, both centrifugal and centripetal. Dr. Arnold also thinks that the cutaneous (sensitive) nerves have this power of conduction in opposite directions. Their centripetal action is acknowledged; the contraction of the skin during various kinds of emotions is due probably to their centrifugal conduction.

We think there is considerable ingenuity and possibly some truth in Dr. Arnold's views, but they are not so clearly stated as is desirable, nor considered in their various relations. If the posterior roots are cutaneous only, and the anterior muscular only, and also insensible to external irritants, how are we to understand the suffering usually consequent on inflammation of tendinous structures? And of what class are the nerves supplied to them? These must come from the sympathetic, as no other origin is possible according to Dr. Arnold's views.

Dr. Arnold is manifestly a man of quick perception and independent temper. He blames his countrymen for the facility with which they receive theories of foreign growth without due examination of the premises, and he cares not to differ from the majority, and loudly to proclaim this difference. This conduct has apparently stirred up a nest of wasps,—small people who wish to recommend themselves to men of influence by a reckless partisanship—gefallsüchtige schwänzeleyen, or in our homely vernacular, fawning tail-waggers. It is quite true that fawning hounds when in packs are very bold and obstreporous; but they obey a master-mind. Let Dr. Arnold give proof of his mastery, not by pulling other men's theories down, but by building up a masterpiece of his own. A true prophet may win honour even in his own country.

